# International Consensus on Guiding Recommendations for Management of Patients with Nonsteroidal Antiinflammatory Drugs Induced Gastropathy-ICON-G

**DOI:** 10.5005/jp-journals-10018-1281

**Published:** 2019-02-01

**Authors:** Richard Hunt, Leonid B Lazebnik, Yury C Marakhouski, Mircea Manuc, Ramesh GN, Khin S Aye, Dmitry S Bordin, Natalia V Bakulina, Baurzhan S Iskakov, Abror A Khamraev, Yurii M Stepanov, Reidwaan Ally, Amit Garg

**Affiliations:** 1 Department of Medicine, McMaster University Health Science Centre, Hamilton, Ontario, Canada; 2 Hospital Therapy, Moscow State University of Medicine and Dentistry, Moscow, Russian Federation; 3 Department of Gastroenterology and Nutrition, Belarusian Medical Academy of Postgraduate Education, Minsk, Belarus; 4 Clinic of Gastroenterology and Hepatology, Fundeni Clinical Institute, Bucharest, Romania; 5 Centre of Excellence in Gastroenterology and Integrated Liver Care Aster Medi City, Cochin, Kerala, India; 6 Department of Gastroenterology, University of Medicine, Yangon, Yangon Region, Myanmar; 7 Department of Pancreatic, Biliary tract and Upper GI disease, A.S. Loginov Moscow Clinical Scientific Center, Moscow, Russian Federation; 8 Department of Therapy and Clinical Pharmacology, North-Western State Medical University, Sankt-Peterburg, Russian Federation; 9 Department of Healthcare, Almaty Health Authority, Almaty, Almaty Province, Kazakhstan; 10 Department of Gatroenterology, Tashkent Medical Academy, Tashkent, Tashkent Province, Uzbekistan; 11 Institute of Gastroenterology of National Academy of Medical Sciences of Ukraine, Dnipropetrovsk Dnipropetrovsk Oblast, Ukraine; 12 Department of Gastroenterolgy, Wits University, Johannesburg, Gauteng, South Africa; 13 Department of Emerging Markets, Dr Reddy’s Laboratories Ltd, Hyderabad, Andhra Pradesh, India

**Keywords:** Gastropathy, Gastroprotective agents, Non-prescription drugs, Nonsteroidal Anti-inflammatory Agents, Proton pump inhibitor.

## Abstract

**Introduction:**

Nonsteroidal anti-inflammatory drugs (NSAIDs), one of the most commonly used medications worldwide, are frequently associated with gastrointestinal adverse events. Primary care physicians often face the challenge of achieving adequate pain relief with NSAIDs, while keeping their adverse events to a minimum. This is especially true when long-term use of NSAIDs is required such as in patients with osteoarthritis and rheumatoid arthritis. To help primary care physicians deal with such challenges more effectively, a panel of expert gastroenterologists came together with the aim of developing practice recommendations.

**Methods:**

A modified ‘Delphi’ process was used to reach consensus and develop practice recommendations. Twelve gastroenterologists from nine countries provided their expert inputs to formulate the recommendations. These recommendations were carefully developed taking into account existing literature, current practices, and expert opinion of the panelists.

**Results:**

The expert panel developed a total of fifteen practice recommendations. Following are the key recommendations: NSAIDs should be prescribed only when necessary; before prescribing NSAIDs, associated modifiable and non-modifiable risk factors should be considered; *H. pylori* infection should be considered and treated before initiating NSAIDs; patients should be properly educated regarding NSAIDs use; patients who need to be on long-term NSAIDs should be prescribed a gastroprotective agent, preferably a proton pump inhibitor and these patients should be closely monitored for any untoward adverse events.

**Conclusion/clinical significance:**

These practice recommendations will serve as an important tool for primary care physicians and will guide them in making appropriate therapeutic choices for their patients.

**How to cite this article:** Hunt R, Lazebnik LB, Marakhouski YC, Manuc M, Ramesh GN, Aye KS, Bordin DS, Bakulina NV, Iskakov BS, Khamraev AA, Stepanov YM, Ally R, Garg A. International Consensus on Guiding Recommendations for Management of Patients with Nonsteroidal Anti-inflammatory Drugs Induced Gastropathy-ICON-G. Euroasian J Hepatogastroenterol, 2018;8(2):148-160.

## BACKGROUND

Nonsteroidal anti-inflammatory drugs (NSAIDs) are used by over 30 million people daily across the globe.^1^ The use of NSAIDs has become widespread due to the availability of these agents both as prescription and as over-the-counter (OTC) medicines.^2^ Even though NSAIDs have proven efficacy in managing pain, fever, and inflammation, they are frequently associated with several untoward adverse events (AEs).^3^ While NSAIDs are also associated with renal (fluid retention, hyper-kalemia, secondary hypertension) and cardiovascular (vascular events, hypertension) AEs, the most common are gastrointestinal (GI) complications, which include-gastritis, ulcers, perforation, and enteropathy.^3,4^A study from Denmark reported an increase in the prevalence of NSAID-related peptic ulcers from 39% in 1993 to 53% in 2002.^5^ Further, the point prevalence of GI complications related to NSAID exposure in the Indian subcontinent in the year 2014 was found to be 30%.^6^ Overall, mortality in patients suffering from an upper gastrointestinal (UGI) bleed or perforation related to NSAIDs use is estimated to be about 1 in 5.^7^ Additionally, the prevalence of gas-troduodenal ulcers in patients taking low-dose aspirin (LDA) is about 10%.^8^

The NSAID-induced gastropathy (NIG) develops at doses that inhibit prostaglandin production, enhance gastric motility, and increase mucosal permeability. This results in neutrophil infiltration and free radical production, and eventually mucosal lesions.^9^ Risk factors include increasing age, the presence of comorbidities especially a history of peptic ulcer disease, liver cirrhosis, and cardiovascular disease. Long-term use of NSAIDs, *Helicobacter pylori (H. pylori)* infection, smoking, chronic alcohol abuse, and concomitant use of other medications increase the risk of developing NIG.^3,10-13^

The NSAIDs have remained the first-line for controlling pain and inflammation particularly in patients with osteoarthritis (OA). Complete withdrawal from NSAIDs is not always practical, particularly in patients with chronic musculoskeletal disorders. Therefore, it is important that clinicians prescribe NSAIDs wiselyto ensure maximum benefits and minimize AEs. All healthcare practitioners, particularly primary care physicians (PCP) can reduce the risk of NIG by careful patient assessment and identification of the risk factors before prescribing an NSAID, educating patients against the addition of OTC NSAIDs, using selective cyclooxygenase-2 (COX-2) inhibitors as first-line medications where appropriate,and co-therapy with a gastroprotective agent (GPA).^1,3,9^

Several international and regional guidelines have been developed to manage NSAID-induced GI complications.^10-17^ However, none specifically focus on management of NIG highlighting the need for a comprehensive clinical guidelineto guide PCPs in the management of NIG, particularly in resource-limited regions of the world. This article presents practice recommendations primarily targeted towards primary care providers, for prevention, early detection, and management of NIG formulated at a meeting held in Dubai, UAE on December 1st, 2016.

## OBJECTIVE

The objective of this consensus meeting was to identify the advances in disease management and the opportu-nitiesfor prevention and management of NIG in nine nations. Further, we attempted to develop definitive clinical practice guidelines for the management of patients with NIG based on the existing literature, real-world evidence, and evidence-based practice.

Ameeting was held before the International Congress of GI Experts, Gastrosphere 2.0 (in Dubai, UAE) in association with the healthy stomach initiative (HSI). The committee of experts from nine nations was named the ICON-G group. Expert representatives proposed recommendations for use by PCPs and internists in the prevention, identification, and management of NIG.

## METHODS

**Fig. 1:**
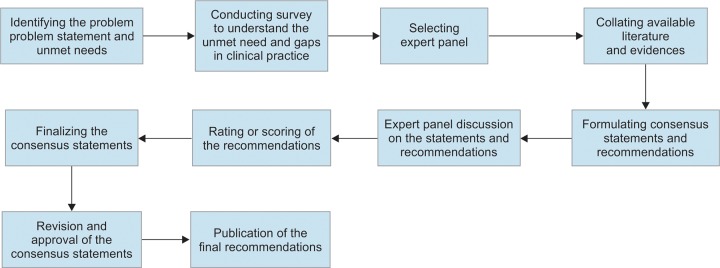
Modified Delphi protocol for consensus development

A modified Delphi consensus process (Fig. 1) was implemented to develop the recommendations.^18,19^ Literature was searched to provide evidence, and recommendations were developed by combining evidence-based and expert consensus-based approach. A comprehensive methodology and transparency in reporting were adopted to develop these clinical practice recommendations.

The process was conducted in two phases. Phase one included an online survey and literature search. The online survey(on online portal survey monkey. com) included twenty questions to establish the current clinical practice in the nine countries. The responses collected from the survey were then used to quantify theknowledgeand practice gap in each of the countries.

An electronic literature search was conducted in PubMed and MEDLINE. The search strategy was developed by combining Medical Subject Headings (MeSH) and free-text keywords using Boolean operators (’OR’ and ‘AND’). Keywords used were “nonsteroidal antiinflammatory drugs”, “NSAIDS”, “gastropathy”, “gastritis”, “ulcers”, “gastric bleeding”, “gastric complications”, and “gastroprotective agents”. Relevant literature from published clinical studies, narrative reviews, systematic reviews, and meta-analyses was collected through September 2016. No additional filters were used during the search. An extensive manual search of literature references was also done, and relevant articles retrieved. Several published international and regional clinical practice guidelines on the management of GI complications were also retrieved.^10-17^All the available literature and evidence were collated and developed into practice recommendations. These were discussed at the meeting and categorized according to the level of evidence.

In phase two, core-committee of ICON-G members comprising of a chairperson and twelve expert gastroen-terologists from Russia, India, Romania, Ukraine, South Africa, Kazakhstan, Belarus, Uzbekistan, and Myanmar, with knowledge and experience of NSAID-induced GI injury, was formed. A pre-meeting draft depicting the purpose of the guidelines, the target patient population, clinical outcomes of interest, key features of the systematic literature review, and a proposed timeline for guideline completion was shared with all panel members. An advisory board meeting chaired by Professor Richard Hunt was conducted with the core-committee of ICON-G on December 1st, 2016 in Dubai (UAE), to discuss and establish the process for the consensus guideline development.

On December 3rd, 2016, the members of Consensus Group (i.e., members of core-committee of ICON-G together with delegates attending the International Congress of GI Experts) discussed and rated their agreement for each recommendation on a four-point Likert scale. Open discussion was conducted for each recommendation, considering evidence and rationale. Voting then took place. About 60% of the voters were gastroenterologists, and 40% were general physicians , with extensive experience of delivering patient care across a wide range of specialties relevant to primary care. The core-committee determined the strength of agreement for each recommendation as follows:

- * Strong Consensus:* If ≥90% of respondents rated the recommendation as “strongly agree” and/or “agree”
- *Consensus:* If ≥80% and <90% of respondents ratedthe recommendation as “strongly agree” and/or “agree”
- *No Consensus:* If <80% of the respondents rated the recommendation as “strongly agree” and/or “agree”

## PRACTICE RECOMMENDATIONS

### Practice Recommendation 1

The NSAID prescription is associated with a high-risk of NIG.

(Consensus: Vote; strongly agree 76%, agree 24%)

### Rationale

Studies have shown that NSAIDs are associated with gastropathy irrespective of the duration of use.

The evidence study (N = 4144) was the largest prospective study of the real-life management of European patients treated with NSAIDs for rheumatic diseases, who wereat an increased GI risk due to advanced age (>60 years), history of peptic ulcer, or concomitant therapy (corticosteroids, anticoagulants).^20^ The study found that NSAIDs were associated with an incidence of 18.5 per 100 person-years for uncomplicated GI events and 0.7 per 100 person-years for complicated GI events. During a median follow-up of 6 months, UGI events (12%) were more common than the lower GI events (1%).^20^

A systematic review (N = 19841) reported that the risk of developing serious NSAID-related GI events is essentially constant over time.^21^ A large autopsy series on patients with a history of NSAID use found a slightly higher number of nonspecific ulcers in the small intestine in long-term NSAID users, and a slightly higher number ofgastric ulcersin short-term NSAID users when compared to long-term users.^22^

A nested case-control study found that, compared to non-users, current NSAID users are at a higher risk of developing serious UGI complications by a factor of 3.7 [95% confidence interval (CI ), 3.1-4.3], and selective COX-2 inhibitors by a factor of 2.6 (95% CI, 1.9-3.6).^23^ This study also foundthat short- and long-term NSAIDs use was associated with a similar risk of UGI complications. Similar findings were reported inother meta-analyses where the relative risk (RR) of serious GI complications was found to be 3 to 4 fold higher in NSAID users compared with non-users irrespective of the duration of use.^24-26^

A multicenter study (N = 187) reported a high point prevalence rate for ulcers (10.7%) and erosions (63.1%) in patients taking LDA. After a follow-up of 3 months (N = 113) the incidence of ulcers anderosions was 7.1% and 60.2%, respectively, suggesting ulcers developed in almost 1 in 10 LDA users.^27^A meta-analysis (N~66000) to assess the incidence of GI hemorrhage associated with aspirin therapy showed that long-term (≥12 months) aspirin treatment was associated with a significant increase in the incidence of GI bleeding. Bleeding was reported in 2.47% of patients taking aspirin compared with 1.42% taking a placebo [odds ratio (OR) 1.68; 95% CI,1.51-1.88].^28^

### Practice Recommendation 2

All NSAIDs, irrespective of their route of administration, have the potential to NIG.

*(Consensus:* Vote; strongly agree 64%, agree 34%, disagree 2%)

### Rationale

Although topical NSAIDs are generally safer than oral NSAIDs withfewer severe GI AEs, 17.5% of patients receiving topical NSAIDs in onestudy reported systemic AEs. Topical NSAIDs potentiated warfarin in five cases, leading to GI bleeding in one case.^29^

A meta-analysis comparing the safety profile of different types of NSAIDs confirmed the variability inRR among various NSAIDs.^30^ Irrespective of the type and formulation, all NSAIDs were associated with GI AEs. The lowest RR was observed with aceclofenac, celecoxib, and ibuprofen, and the highest with piroxicam, ketoro-lac, and azapropazone. Rofecoxib, sulindac, diclofenac, meloxicam, nimesulide, ketoprofen, tenoxicam, naproxen, indomethacin, and diflunisal were associated with moderate risks.^30^ In another meta-analysis, nabumetone was associated with a very low GI risk when compared to other NSAIDs (p = 0.007).^31^

Somenovel NSAID formulationsare safer compared to the conventional forms. A meta-analysis of randomized controlled trials (RCTs) concluded that AMG is an effective anti-inflammatory drug with an improved GI tolerability profile, and lower incidence of GI AEs compared to traditional NSAIDs. The endoscopy scores were higher for other NSAIDs than AMG (27.6% versus 21.4%; p < 0.05). Likewise, post-treatment severity of gastric and/ or duodenal ulcer was higher in the NSAID group than AMG group (14.2% *vs.* 4.3%; p<0.05).^32^

### Practice Recommendation 3

The most common non-modifiable risk factors for NIG includeage (>60 years), a prior history of peptic ulcers, and complications such asa history of gastrointestinal bleeding.

*(Consensus:* Vote; 65% strongly agree, 33% agree, 2% disagree)

### Rationale

Age is one of the strongest predictors of NSAID related GI complications. A large prospective multicenter study in rheumatoid arthritis (RA) patients (N = 2747) found that the principal risk factors (p < 0.05) were increasing age, a history of NSAID-related ulcer and its complications, and corticosteroid use.^33^ Similarly, another study reported that age >60 years (OR, 5.52; 95% CI, 4.63-6.60) and previous history of GI complications (OR, 4.76; 95% CI, 4.05-5.59)were associated with an increased risk for serious GI AEs.^21^

Another study reported that the risk for serious NSAID induced GI eventswas nearly doubled with each of the following factors: a history of previous GI events, peptic ulcer, concomitant glucocorticoid treatment, and severe arthritis induced disability. Using multiple drugs, changing the NSAID, or using high dosesincreasedthe risk by at least 6-fold. Age > 65 years and a history of cardiovascular disease increased the risk by 2 to 3 fold. The presence of multiple risk factors also increased the incidence of GI complications during NSAID therapy.^34^

Although increasing age is a major risk factor for NSAID-induced GI complications, these problems are also seen in the pediatric population. In a retrospective, multicenter study of Italian children (N = 51) attending the emergency unit with GI bleeding following NSAIDs use, 62% of patients had endoscopically confirmed gastric lesions, 33% had duodenal lesions, and 15% had esophageal lesions. Of particular concern, 6% required endoscopic hemostasis to control GI bleeding. These findings emphasize that children are also vulnerable to NSAID-induced gastric complications.^35^

### Practice Recommendation 4

The modifiable risk factors for NIG include concomitant use of aspirin and/or other NSAIDs, systemic corticosteroids, anticoagulants/antiplatelet, and selective serotonin reup-take inhibitors (SSRIs).

*(Consensus:* Vote; strongly agree 59%, agree 37%, disagree 4%)

### Rationale

Results from the Italian pharmaco vigilance reporting system show that the combined use of LDA and another NSAID or use of multiple NSAIDs is associated with an increased incidence of GI adverse events.^36^ Further, the Spanish safe prescription recommendations emphasize that two or more NSAIDs simultaneously do not increase effectiveness but do increase toxicity.^37^

A meta-analysis summarized the results from 16 studies reporting an almost twofold increase in the risk of serious GI complications when NSAIDs were used concomitantly with corticosteroids compared to NSAIDs alone (OR, 1.83; CI, 1.20-2.78).^21^

A case-control study from the National Health System in Spain reported that concomitant use of NSAIDs with clopidogrel/ticlopidine (RR, 15.2; 95% CI, 4.1-56.5)oranti-coagulants (RR, 19.3; 95% CI 8.2-45.3) increased the risk of UGI bleeding substantially.^38^ Similarly, in another case-control study from the United Kingdom General Practice Research Database, combining NSAIDs with clopidogrel (RR, 2.93; 95% CI, 1.74-4.93) or warfarin (RR, 4.60; 95% CI, 2.77-7.64) was associated with an increased risk of GI bleeding.^39^ Another study found that combined use of an NSAID with LDA (OR, 4.3; 95% CI, 1.7-11; p < 0.01) or other antiplatelet drugs (OR, 4.9; 95% CI, 1.4-17; p = 0.01) was associated with a greater risk of lower GI bleeding than when used alone (OR, 2.3; 95% CI, 1.6-3.2; p < 0.01). Furthermore, a combination of NSAIDs was associated with a higher risk than use of a single NSAID (OR, 4.9; 95% CI, 2-12; p < 0.01).^40^

A population-based retrospective cohort study reported that there could be up to a 50% increase in the risk of GI bleeding with dabigatran compared with warfarin and a more than the twofold higher risk of bleeding with rivaroxaban compared with warfarin.^41^ Combining these newer antiplatelet drugs with NSAIDs carries a definite increased GI risk of bleeding.

Selective serotonin reuptake inhibitors may also lead to GI bleeding when given with NSAIDs, by impairing the metabolism of NSAIDs leading to an increase in their blood levels,and also by inhibiting hemostasis.^42,43^A systematic review showed that the RR for UGI bleeding from NSAID and SSRI combination compared to use of neither drug was 3.3-15.6 and that for GI AEs was 12.4.^44^ A case-control study showed a moderate increase in UGI risk with concurrent use of SSRIs and NSAIDs when com pared to NSAIDs use alone (OR, 1.57; 95% CI, 1.24-1.99) and higher increase, when compared to use of neither drug (OR, 4.19; 95% CI, 3.30-5.31).^45^ Similar results, were found in several other studies where the combined use of NSAIDs and SSRIs was associated with an OR of about 4 for GI AEs.^46,47^

### Practice Recommendation 5

*H. pylori* infection increases the risk of developing NIG. It is suggested that physicians consider the possibility of *H. pylori* infection and treat it, if present, prior to prescribing NSAIDs.

*(Consensus:* Vote; strongly agree 58%, agree 38%, disagree 4%)

### Rationale

*H. pylori* infection together with the use of NSAIDs is a well-known risk factor that induces gastroduodenal mucosal damage and ulcers.^48^A meta-analysis found that NSAIDs and *H. pylori* infection are not only independent risk factors, but they also have a synergistic effect on the development of peptic ulcer and ulcer bleeding.^49^ While the risk of ulcer bleeding increased 1.79-fold with *H. pylori* infection and 4.85-fold with NSAID use, the two together increased the risk 6.13-fold.^49^ In a systematic review of 21 studies (N = 10146), the peptic ulcer was more common in NSAID users who were *H. pylori* positive (OR, 1.81; 95% CI, 1.40-2.36).^50^ Furthermore, in a meta-analysis of RCTs, 7.4% of patients developed an ulcer in the *H. pylori* eradicated group compared with 13.3% in the control group (OR, 0.43; 95% CI, 0.20-0.93).^51^A recent retrospective study of 245 patients taking an NSAID or LDA continuously for at least 3 months reported that presence of *H. pylori* infection increases the risk of severe gastric mucosal injury (OR, 2.0; 95% CI, 1.2-3.5).^52^

All these studies highlight that NSAIDs and *H. pylori* infection synergistically cause gastric mucosal injury. However, *H. pylori* testing may not be practical for all patients especially in the primary care setting.^53^ Testing must be carried out when the patient is prescribed long-term NSAIDs in areas where *H. pylori* are common. In current practice, *H. pylori* eradication therapy comprises of PPIs and antimicrobials agents, including bismuth compounds, clarithromycin, amoxicillin, metronidazole, levofloxacin, furazolidone, doxycycline, nitazoxanide, andrifabutin.^54,55^

### Practice Recommendation 6

It is suggested that physicians consider prescribing NSAIDs only when indicated.

The prescribing physician should consider patient-specific risk factors before prescribing NSAIDs.

*(Consensus:* Vote; strongly agree 69%, agree 29%, disagree 2%)

### Rationale

Clinicians must identify NSAIDs use as a risk factor for GI complications and initiate preventive treatment. A population-based cohort study showed that despite the risk of UGI events, guidelines for GPA use were followed in less than half of the cases. GPAs were prescribed in 31.8% of high-risk patients receiving LDA and 48.0% of those receiving NSAIDs.^56^ Similarly, the *Canadian Osteoarthritis* Rx (CANOAR) study examined NSAID use in clinical practice in a cohort of PCPs and compared it with the osteoarthritis treatment guidelines. Overall, 58% of the prescriptions were found to be appropriate considering the GI risk of the patients.^57^ Thus, there is an opportunity for improvement in implementing strategies by adherence to guidelines.^56,57^

A RCT evaluated a physician education program, communicating OA management guidelines in elderly patients, which emphasized avoidance of NSAIDs. Physicians (N = 209) were visited with reminders for a re-evaluation of their patients’ NSAIDs. Brief physician educational visits resulted in a 7% (95% CI, 3-11%) reduction in NSAIDs use without undesirable substitution of other medications or detectable worsening of musculo-skeletal symptoms.^58^

A Korean observational study of NSAID prescription patterns in orthopedic patients highlighted the need for better understanding of patient-specific risk factors among prescribing physicians. The study showed that, despite being identified as high-risk or at very high GI risk (by a risk scoring scale), only 51% of patients were given a COX-2 selective inhibitor instead of a traditional NSAID. Physician’s preference for a particular NSAID and not considering a patient’s GI risk factors may expose patients to increased risk of NSAID-induced GI compli-cations.^59^

### Practice Recommendation 7

It is recommended that the prescribing physician educates the patient against self-medication with NSAIDs and the importance of regular follow-up, particularly in those on long-term and/or high-dose NSAIDs.

*(Consensus:* Vote; strongly agree 54%, agree 41%, disagree 4%)

### Rationale

When the prescribed NSAIDs do not adequately control pain, patients may often seek additional OTC NSAIDs.^60^ They may not be aware that OTC pain-relief medications belong to the same class as their prescribed NSAIDs, resulting in high dose NSAID treatment. Patients may thus increase their risk of developing GI complications. In one study, total 26% of the participants were users of two NSAIDs and had poorer health-related quality of life (QoL) compared to those not on high doses of NSAIDs.^60^ A cross-sectional study, conducted in the Netherlands, assessed the prevalence of OTC-NSAID use in a sample of the general population (N = 118) and in a sample of patients (N = 264) with a high risk of developing serious NSAID-related AEs.^2^ The results of this study showed high and unregulated use of NSAIDs. OTC NSAIDs were used by 30% of the general population and 13% of the high-risk sample. OTC NSAIDs dose exceeded the recommended daily maximum by 9% and 3% in the general population and high-risk sample respectively.^2^ Proper patient education, is, therefore, of the utmost importance.

Patients must also be informed about the importance of adherence to GPAs. One study evaluated the association between adherence to GPA and UGI events among NSAID users. Among those who were non-adherent, the OR was 2.39 (95% CI, 1.66-3.44) for all UGI events and 1.89 (95% CI, 1.09-3.28) for UGI bleeding alone, compared to those who adhered fully, emphasizing the importance of implementing strategies to follow and improve GPA adherence.^61^

### Practice Recommendation 8

In patients on long-term NSAID treatment, regular and patient-specific monitoring (with due consideration to comorbidities, other medications, and the presence of all risk factors) is recommended for evidence of gastrointestinal bleeding and/or gastrointestinal side-effects.

*(Consensus:* Vote; strongly agree 67%, agree 27%, disagree 4%, strongly disagree 2%)

### Rationale

Scarpignato and Hunt (2010) suggested that physicians should not prescribe NSAIDs before taking a careful history and undertaking a physical examination, to evaluate patient-specific risks and benefits for NSAID therapy.^62^ Furthermore, in the presence of GI and/or cardiovascular risk factors, appropriate preventive strategies (i.e., COX-2 selective inhibitors and/or PPI use as well as the need for LDA) should be implemented from the start of treatment with compliance assessed regularly, especially in high-risk patients.^62^

### Practice Recommendation 9

Co-therapy with a PPI is the preferred approach for prevention of NIG. Alternatively, a histamine-2 receptor antagonist (H2RAs) or misoprostolcan be used.

*(Consensus:* Vote; strongly agree 84%, agree 14%, disagree 2%)

### Rationale

A systematic reviewand meta-analysis emphasized the superiority of PPI therapy over H2RA or placebo in reducing mortality among patients with an endoscopi-cally confirmed high-risk of peptic ulcer bleeding. PPI therapy also reduced rates of re-bleeding and the need for surgical intervention.^63^

In a meta-analysis including 10 RCTs (N = 8780), PPIs reduced the risk of LDA-associated UGI ulcers (OR = 0.16; 95% CI, 0.12-0.23) and bleeding (OR = 0.27; 95%CI, 0.16-0.43) compared with control (placebo, a cytoprotec-tive agent, or an H2RA).^64^

A double-blind RCT (OMNIUM study) compared omeprazole and misoprostol for NSAID-induced ulcers. Although the two drugs were overall equally effective, remission rates were better with omeprazole(61% *vs* 48%; p = 0.001). Moreover, omeprazole was better tolerated than misoprostol.^65^

A RCT comparing misoprostol with two doses of lansoprazole reported that both drugs were equally effective and superior to placebo. After considering the withdrawals due to AEs, treatment was deemed successful in 69% of patients in each treatment group *vs.* 35% for placebo.^66^

A Cochrane review found that misoprostol significantly reduced the RR of gastric ulcer by 74% (RR, 0.26; 95% CI, 0.17-0.39) corresponding to a 12% absolute risk reduction.^67^ The review also reported that, while a standard dose of H2RA was effective at reducing the risk of endoscopic duodenal ulcer (RR = 0.36; 95% CI, 0.18-0.74), a double dose was required toreduce the risk of endoscopic gastric ulcer (RR = 0.44; 95% CI, 0.26-0.74).^67^

### Practice Recommendation 10

There is no difference in effectiveness between different PPIs (esomeprazole, lansoprazole, omeprazole, pantopra-zole, and rabeprazole) available in the market. All available PPIs at recommended doseshave similar efficacy in reducing the risk of NIG.

*(Consensus:* Vote; strongly agree 76%, agree 24%)

### Rationale

Few head-to-head studies are comparing the different PPIs in the managementof NIG. However, the available data suggest that all PPIs are essentially equivalentin the management of NIG. A randomized, double-blind study (N = 595) to compare pantoprazole (20 mg OD and 40 mg OD) withomeprazole (20 mg OD) for the prevention of GI lesions associated with NSAIDs found that both PPIs were similarly effective (remission rates for lack of “therapeutic failure” were 90%, 93%, and 89%, and for lack of “endoscopic failure” were 91%, 95%, and 93% (for pantoprazole 20 mg OD, pantoprazole 40 mg OD, and omeprazole 20 mg OD, respectively).^68^

A systematic review comparing the efficacy of different available PPIs found that for symptom relief in gastroesophageal reflux disease lansoprazole was faster than omeprazole, and esomeprazole was faster than both lansoprazole and omeprazole. However, none of these-drugs was found to be superior over the other.^69^

A few novels or immediate-release formulations of PPIs have also been studied.^70^ The immediate-release omeprazole (Zegerid^®^, Omez-Insta^®^) are associated with faster absorption of omeprazole, the more rapid onset of anti-secretory activity, and a slightly longer duration of acid suppression. This formulation is not food dependent and provides dosing flexibility which is considered apt for suppressing nocturnal gastric acid secretion.^71^ Immediate-release omeprazole has also shown superiority over once daily dosing of delayed-release PPIs in control of nocturnal gastric acidity.^72^ Dexlansoprazole is a PPI with adual delayed-release formulation, which produces a dual-peak pharmacokinetic profile unlike the single peak profile of the first generation or delayed release PPIs. Thus, dexlansoprazole maintains therapeutic plasma drug concentrations longer than lansoprazole and other PPIs.^73^

### Practice Recommendation 11

It is suggested that physicians consider continued prophylaxis with a PPI when NSAIDs are prescribed.

*(Consensus:* Vote; strongly agree 69%, agree 31%)

### Rationale

In a large international multicenter study (N = 610), both omeprazole and misoprostol improved QoL in chronic NSAID users with NSAID-associated gastroduodenal lesions. However, omeprazole relieved gastrointestinal symptoms better than misoprostol. Hence, it is appropriate to co-prescribe a PPI in this patient group for prophylaxis as long as NSAIDs are prescribed and taken.^74^

Long-term PPI treatment is associated with better-outcomes in patients continuing NSAID therapy. A large RCT in OA and RA patients compared omeprazole with ranitidine in the prevention of gastric and duodenal ulcers. Omeprazole healed and prevented ulcers more effectively than ranitidine over 8 weeks of the study. During the subsequent 6-month maintenance treatment, 72% and 59% of patients were in remission in the omepra-zole and ranitidine groups respectively. Relapses were more common with ranitidine than with omeprazole.^75^

In long-term NSAID users, a PPI can substantially reduce the occurrence of GI complications. A 3-month studyto evaluate omeprazole 20 mg every morning as primary prophylaxis against NSAID-induced ulcer and dyspepsia, found that the estimated probability of remaining symptom-free for 6 months for patients taking omeprazole was 0.78 compare d to 0.53 for placebo (p = 0.004).^76^ Thus, continuing a PPI is important in the management of NIG. Although long-term PPIs could be associated with some AEs, in patients with a clear indication for the PPI, the risk-benefit ratio favors PPI use.^43^

### Practice Recommendation 12

The following clinical presentations in a patient taking an NSAID to raise the suspicion of NIG: abdominal cramps/ pain, dyspepsia, nausea, and vomiting.

In patients with pre-existing risk factors for gas-tropathy, it is suggested that the physician maintain a high index of suspicion of onset of NSAID-induced gastropathy in patients on NSAID therapy developing such symptoms.

*(Consensus:* Vote; strongly agree 43%, agree 40%, disagree 9%, and strongly disagree 9%)

### Rationale

Upper GI symptoms including dyspepsia, heartburn, bloating or cramping, nausea, and vomiting are reported in up to 40% of patients taking NSAIDs.^77^ However, in 50 to 60% NSAIDs users, GI complications may be clinically asymptomatic.^78^ Hence, close monitoring is advisable forall patients on long-term NSAIDs, particularly those with the presence of one or more risk factors.

### Practice Recommendation 13

It is suggested that to determine the risk, physicians take a careful history andperform necessary laboratory tests when starting NSAID treatment.

If a patient presents with a history, or clinical symptoms and signs suggesting NSAID-related GI problems, the PCP should consider referring the patient to a specialist for further management.

*(Consensus:* Vote; strongly agree 38%, agree 54%, disagree 16%, and strongly disagree 2%)

### Rationale

Standard approaches for diagnosing NIG include identification of high-risk populations; history, clinical presentation, and examination of the patient; tests for *H. pylori* infection in suspected cases; and hemoglobin and hematocrit tests. Upper GI endoscopy is required to confirm the diagnosis.^79^

### Practice Recommendation 14

While PPIs are the preferred drugs, some choice of GPA (in treatment doses) is recommended for patients diagnosed with NIG.

*(Consensus:* Vote; strongly agree 40%, agree 40%, disagree 17%, and strongly disagree 2%)

### Rationale

Several studies have highlighted the efficacy of PPIs in the treatment of LDA- and NSAID-induced GI injury.^63,64,80-82^ Evidence also indicates that PPIs are superior to other GPAs.^63-65,74,75^ Therefore, PPIs remain the preferred treatment. Nevertheless, other GPAs, including H2RAs and misoprostol, have also demonstrated effectiveness in the treatment of NIG,^66,67,83,84^ and may be considered when PPIs cannot be prescribed.

### Practice Recommendation 15

A fixed-dose combination (FDC) of an NSAID and a GPA is not recommended in patients with NIG.

*(Consensus:* Vote; strongly agree 59%, agree 28%, disagree 13%)

### Rationale

The ketoprofen-omeprazole FDC was the first NSAID GPA combination to be approved.^85^ Other commonly used FDCs include ibuprofen-famotidine and naproxen-esomeprazole.^86,87^ Although FDCs are believed to be associated with better compliance; sufficient clinical evidence is not available to support their superiority regarding efficacy and safety.^86,87^ Furthermore, the high-cost of FDC makes prescription difficult in long-term users, such as patients with OA.

Boxes 1 and 2 list the recommendations for doctors in the treatment of NIG for highrisk and moderate risk patients respectively.

## CONCLUSION

NSAIDs are the most widely used drugs for controlling pain and inflammation. Easy availability (as OTC drugs) and good efficacy of these drugs increase their use. However, the gastrointestinal complications, particularly gastropathy, remain a matter of concern for the prescribing physician. The risks of developing gastropathy further increase with high-dose, long-term, or inappropriate use of NSAIDs especially in the elderly. Hence, recommendations are necessary to guide a PCP in making safe and sensible use of NSAIDs and to minimize the risk of gastropathy. The Consensus Group recommends the use of NSAIDs only as and when indicated. Associated risk factors and a risk profile of the patient is important and should be determined before prescribing NSAIDs. Additionally, physicians are encouraged to take time to educate their patients about the safe use of NSAIDs properly, to avoid dose creep, and not combine their prescribed drugs with OTC medications without prior discussion with their physician. Furthermore, risk monitoring of patients undergoing long-term NSAID treatment and co-therapy with a GPA usually a PPI is also recommended. The consensus group emphasizes the importance of the patient’s age, history, clinical presentation, and presence of *H. pylori* infection for timely management of NIG; and the need to develop clinical strategies to ensure GI safety for patients undergoing NSAID treatment.

**Table:** **Box 1:** Recommendations for doctors-high risk patients

1.		Document patient’s history and carry out necessary laboratory investigations before starting any treatment	
2.		For high-risk patients, avoid NSAIDs and use alternate management strategies like physiotherapy and/or exercise to ease pain and inflammation in diseases like OA	
3.		Consider prescribing low doses and shorter durations for diseases that require instant relief (headache, dysmenorrhea, post-operative pain)	
4.		Consider pulse therapy - prescribing large doses NSAIDs in an intermittent manner to enhance the therapeutic effect and reduce the GI complications/other adverse events in chronic diseases	
5.		Avoid prescribing NSAIDs with other drugs (SSRIs, antiplatelet drugs, corticosteroids)	
6.		Inform the patient that NSAIDs can be taken “as required” and generally do not have a fixed schedule	
7.		Prefer selective COX-2 inhibitors like coxibs or safer drugs like amtolmentinguacyl over conventional NSAIDs	
8.		Prescribe double dose of gastroprotective agents like PPIs in patients undergoing long-term (>30 days) NSAID treatment	
9.		Prescribe a single dose of PPI (up to 30 days) in patients undergoing NSAID treatment	
10.		Perform periodic assessment of patients on long-term NSAID therapy for early identification of signs/symptoms of gastropathy	

Abbreviations: COX-2, cyclooxygenase-2; GI, gastrointestinal; NSAID, non-steroidal anti-inflammatory drug; OA, osteoarthritis; SSRI, selective serotonin reuptake inhibitor

**Table:** **Box 2:** Recommendations for doctors-moderate risk patients

1		Document patient’s history and carry out necessary laboratory investigations before starting any treatment	
2.		Consider prescribing topical NSAIDs over oral NSAIDs in patients experiencing mild pain like muscle pain, or low back pain	
3		Initiate prophylactic therapy with PPI (e.g. Omeprazole 20 mg once daily) to reduce the risk of gastropathy	
4.		Consider *H. pylori* eradication in case it was diagnosed, and eradication therapy not given	
5.		Include an antacid in the drug regimen as antacids neutralize existing stomach acid and can provide rapid pain relief	
6.		Consider probiotic supplement (containing Lactobacillus acidophilus). Probiotics or “friendly” bacteria may help maintain a balance in the digestive system between good and harmful bacteria	
7.		Suggest a multivitamin daily to improve digestive health (e.g. multivitamins containing the antioxidant vitamins A, C, E, the B vitamins, and trace minerals, such as magnesium, calcium, zinc, and selenium)	
8.		Guide patients to avoid self-medication, high dose unregulated use of NSAIDs, and to never combine OTC medications without consultation	
9.		Counsel the patients well and educate them on the signs and symptoms of gastropathy so that they can reach for help at the earliest	
10.		Suggest the patients to visit regularly so that the need for further treatment with NSAIDs can be reviewed	

Abbreviations: COX-2, cyclooxygenase-2; GI, gastrointestinal; NSAID, non-steroidal anti-inflammatory drug; OA, osteoarthritis; OTC, over-the-counter; SSRI, selective serotonin reuptake inhibitor

**Table:** Voting Participants

*Participant Name*		*Country*	
Julia Gorgun		Belarus	
Jan Tack		Belgium	
Richard Hunt		Canada	
G N Ramesh		India	
Almagul Kuzgibekova		Kazakhstan	
Yerlan Bazargaliyev		Kazakhstan	
Gyuzel Jakupova		Kazakhstan	
Baurzhan Iskakov Samikovich		Kazakhstan	
Thein Saw		Myanmar	
Win Phyu Phyu Myint		Myanmar	
Khin San Aye		Myanmar	
Paul Jurgen Porr		Romania	
Elena-Tatiana Ivan		Romania	
Radu-Bogdan Mateescu		Romania	
Cristina Daniela Bura		Romania	
Ligia Ariana Bancu		Romania	
Victor-CatalinSfarti		Romania	
Mircea Manuc		Romania	
Lucian Negreanu		Romania	
Elena Onuchina		Russia	
Mikhail Sheviakov		Russia	
Tatiana Sviridova		Russia	
Oksana Pozdniakova		Russia	
Elina Petrova		Russia	
Svetlana Turkina		Russia	
Elena Vyuchnova		Russia	
AimanSarsenbaeva		Russia	
Irina Kozlova		Russia	
Aleksandr Stepchenko		Russia	
Oleg Mironchev		Russia	
Alexey Okhlobystin		Russia	
Tatiana Iankovaia		Russia	
Elena Li		Russia	
Inna Putintseva		Russia	
Zarina Galeeva		Russia	
Sergei Alekseenko		Russia	
Elena Miguskina		Russia	
EmiliyaYakovenko		Russia	
Elena Kashkina		Russia	
Leonid Lazebnik		Russia	
Natalia Bakulina		Russia	
Victor Pasechnikov		Russia	
Dmitry Bordin		Russia	
Anell Meyer		South Africa	
Reidwaan Ally		South Africa	
Bilal Bobat		South Africa	
Vasudevan Naidoo		South Africa	
Fritz Potgieter		South Africa	
Monique Marais		South Africa	
Hitendrakumar Bhaga		South Africa	
Sandie Thomson		South Africa	
Yurii Stepanov		Ukraine	
Olha Bondarenko		Ukraine	
Andriy Dorofyeyev		Ukraine	
Abror Khamraev		Uzbekistan	
Feruza Khamrabaeva		Uzbekistan	
